# Parallel Olfactory Processing in the Honey Bee Brain: Odor Learning and Generalization under Selective Lesion of a Projection Neuron Tract

**DOI:** 10.3389/fnint.2015.00075

**Published:** 2016-01-19

**Authors:** Julie Carcaud, Martin Giurfa, Jean Christophe Sandoz

**Affiliations:** ^1^Evolution, Genomes, Behavior and Ecology, Centre National de la Recherche Scientifique, Univ Paris-Sud, IRD, Université Paris-SaclayGif-sur-Yvette, France; ^2^Research Center on Animal Cognition, Université Toulouse III - Paul SabatierToulouse, France; ^3^Research Center on Animal Cognition, Centre National de la Recherche ScientifiqueToulouse, France

**Keywords:** insect, olfaction, parallel processing, projection neurons, specific lesion, olfactory conditioning

## Abstract

The function of parallel neural processing is a fundamental problem in Neuroscience, as it is found across sensory modalities and evolutionary lineages, from insects to humans. Recently, parallel processing has attracted increased attention in the olfactory domain, with the demonstration in both insects and mammals that different populations of second-order neurons encode and/or process odorant information differently. Among insects, Hymenoptera present a striking olfactory system with a clear neural dichotomy from the periphery to higher-order centers, based on two main tracts of second-order (projection) neurons: the medial and lateral antennal lobe tracts (m-ALT and l-ALT). To unravel the functional role of these two pathways, we combined specific lesions of the m-ALT tract with behavioral experiments, using the classical conditioning of the proboscis extension response (PER conditioning). Lesioned and intact bees had to learn to associate an odorant (1-nonanol) with sucrose. Then the bees were subjected to a generalization procedure with a range of odorants differing in terms of their carbon chain length or functional group. We show that m-ALT lesion strongly affects acquisition of an odor-sucrose association. However, lesioned bees that still learned the association showed a normal gradient of decreasing generalization responses to increasingly dissimilar odorants. Generalization responses could be predicted to some extent by *in vivo* calcium imaging recordings of l-ALT neurons. The m-ALT pathway therefore seems necessary for normal classical olfactory conditioning performance.

## Introduction

In many sensory modalities, the nervous system uses parallel pathways to enable the separate processing of different stimulus features. The best described example of such parallel stimulus segregation is the case of visual processing in vertebrates (Goodale et al., 1994) and invertebrates (Strausfeld and Lee, 1991; Yamaguchi et al., 2008) which relies on the existence of one pathway involved in the processing of colors and shapes and of another pathway processing movement and spatial features (Ettlinger, 1990). The study of such parallel processes usually follows a double approach: (i) functional recording (via electrophysiology or imaging, for instance) of individual responses from each of these pathways (Stecker et al., 2003) and (ii) selective pathway lesions allowing to determine the capabilities affected by the injury and thus the functional role of both the lesioned and the intact pathways (Lomber and Malhotra, 2008; Strutz et al., 2014). Combining both approaches is essential for understanding parallel processing in a given sensory system.

In the olfactory modality, parallel processing is least known, although the anatomical organization of olfactory systems clearly suggests that such treatment exists (Breer et al., 2006; Galizia and Rössler, 2010; Fukunaga et al., 2012; Igarashi et al., 2012; Rössler and Brill, 2013). Both in vertebrates (Spehr et al., 2006) and insects (Mustaparta, 1996; Hansson and Anton, 2000), different subsystems are involved in the processing of pheromones and general odorants. Besides this segregation in terms of odorant classes, the general olfactory system needs to classify the chemical quality of odorants regardless of their concentration (“concentration invariance”) and also code the absolute concentration of an odor when an animal seeks its source (Uchida and Mainen, 2008; Asahina et al., 2009). In addition, different chemical characteristics of odorant molecules (for instance their chain length or functional group) may need to be processed separately. Parallel processing in the olfactory system may constitute an adequate solution to these problems. However, how parallel olfactory systems encode and process chemical stimuli is still largely unknown (Breer et al., 2006; Nawrot, 2012; Rössler and Brill, 2013).

**Figure 1:**
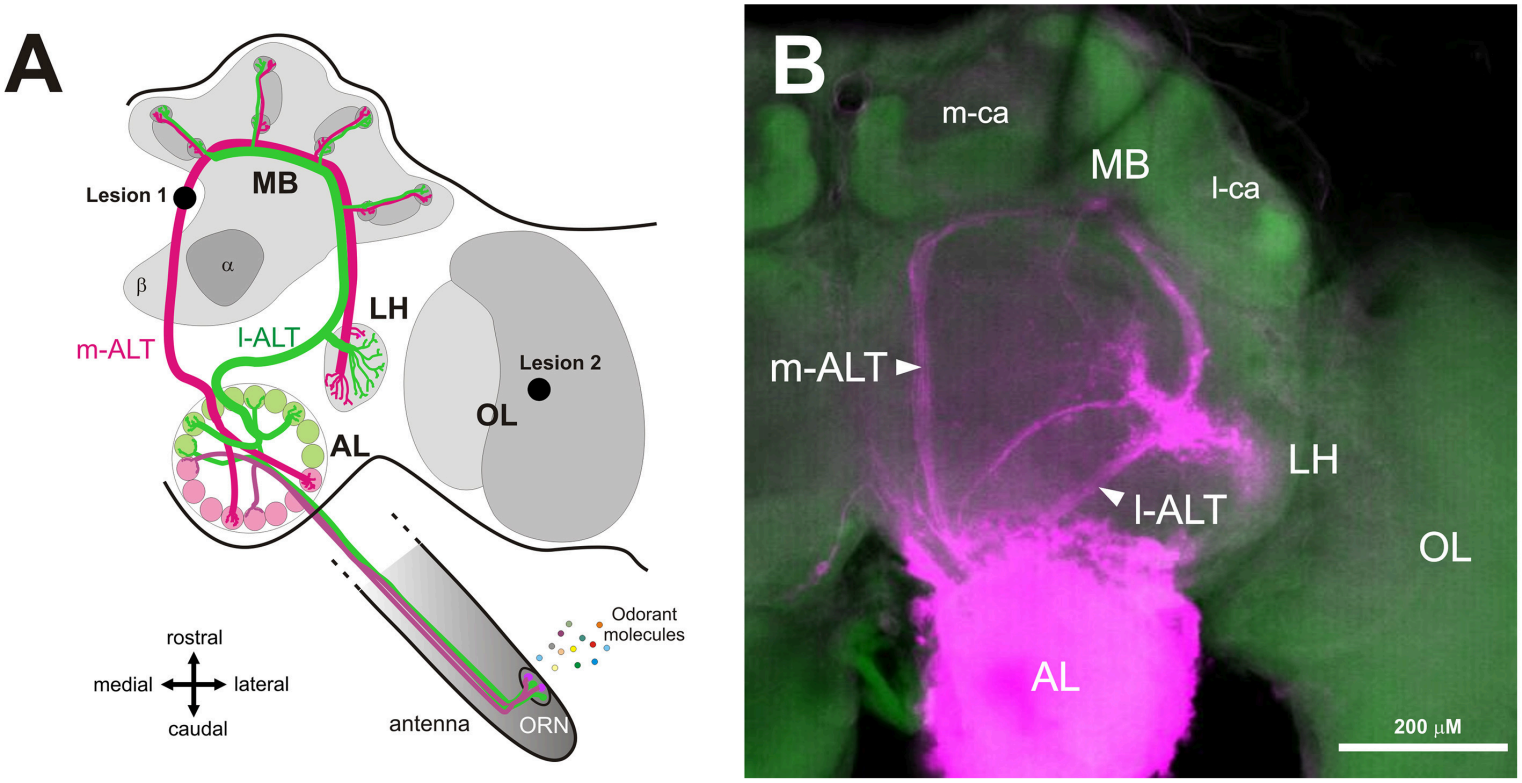
**Dual olfactory pathway of the honey bee brain. (A)** Schematic overview of the dual olfactory pathway of the honey bee brain (adapted from Carcaud et al., 2012, 2015). Odorant molecules are detected by olfactory receptor neurons (ORN) on the antenna which project to the antennal lobe (AL). Then, projection neurons (PN) convey information to the mushroom bodies (MB) and the lateral horn (LH) via two main tracts, the medial antennal lobe tract (m-ALT, magenta) and the lateral antennal lobe tract (l-ALT, green). Lesion site of the m-ALT and of the optic lobe (OL) are indicated (lesion 1 and lesion 2 respectively). **(B)** Mass staining in the AL, showing the course of l-ALT and m-ALT PNs from the AL to LH and the MB calyces. Abbreviations: m-ca, median calyx; l-ca, lateral calyx.

The honey bee *Apis mellifera* is an influential model for the study of olfactory coding and processing. Olfaction is a key modality for honey bees, playing a major role in multiple aspects of their social life style (Free, 1987; Sandoz et al., 2007) and foraging behavior (von Frisch, 1967; Menzel, 1999; Giurfa, 2007). The olfactory circuit of the bee exhibits two parallel olfactory pathways of almost equal size (Abel et al., 2001; Kirschner et al., 2006; Galizia and Rössler, 2010; Rössler and Brill, 2013). Following odor detection by olfactory receptor neurons (ORNs) and subsequent primary processing in the antennal lobe (AL), two main neural tracts of projection neurons (PNs), the lateral and the medial antennal lobe tracts (l-ALT and m-ALT, respectively) convey the processed olfactory message to higher-order centers, the mushroom bodies (MBs) and the lateral horn (LH; Figure 1). The AL is composed of functional units, termed glomeruli, that each receives input from ORNs expressing the same olfactory receptor type (Vosshall et al., 2000). About half of the glomeruli located on the ventral surface of the AL (84 glomeruli) are innervated by the l-ALT while the other half located on the dorsal surface (77 glomeruli) are innervated by the m-ALT. The two tracts project to largely segregated areas within higher-order centers, with only limited overlap (Kirschner et al., 2006). Until now, only functional recordings have been used in the honey bee to study the role of these parallel pathways but no clear differences were found in their responses to general odorants, which are mostly redundant apart from small disparities in their spatiotemporal characteristics (Müller et al., 2002; Krofczik et al., 2009; Yamagata et al., 2009; Carcaud et al., 2012; Brill et al., 2013, 2015). The most apparent difference between both tracts was the fact that queen pheromone is processed by the l-ALT while brood pheromone is mainly processed by the m-ALT (Carcaud et al., 2015). Apart from these differences, the two pathways may also be differentially involved in olfactory learning, but this idea has not been explicitly tested. In this context, the use of selective tract lesions may help understand the functional role of l-ALT and m-ALT neurons.

In honey bees, classical conditioning of the proboscis extension response (PER) is commonly used for studying olfactory perception and learning (Smith and Menzel, 1989; Getz and Smith, 1991; Sandoz et al., 2001; Guerrieri et al., 2005). In this protocol, bees learn to associate an initially neutral odor (conditioned stimulus—CS) with a sucrose reward (unconditioned stimulus—US) applied to the antennae and then to the proboscis (Bitterman et al., 1983; Giurfa and Sandoz, 2012). Following conditioning, bees extend their proboscis in response to the odor alone (Takeda, 1961; Bitterman et al., 1983). The aim of this work was to combine specific PN lesions with olfactory PER conditioning. Because of the close proximity of the l-ALT with the path of the VUM-mx1 neuron, which is known to represent the appetitive sucrose reinforcement in the bee brain and is thus critical for appetitive conditioning (Hammer, 1993), only lesions of the m-ALT pathway could be applied. We thus damaged the m-ALT between the AL and its upstream targets (MB and LH). Bees were then subjected to an olfactory conditioning procedure followed by generalization tests, using a range of odorants differing in carbon chain length and/or functional group, features that affect odorant similarity. Our data suggest that m-ALT neurons are necessary for supporting normal olfactory learning acquisition performance.

## Materials and methods

### Honey bee preparation

**Figure 2:**
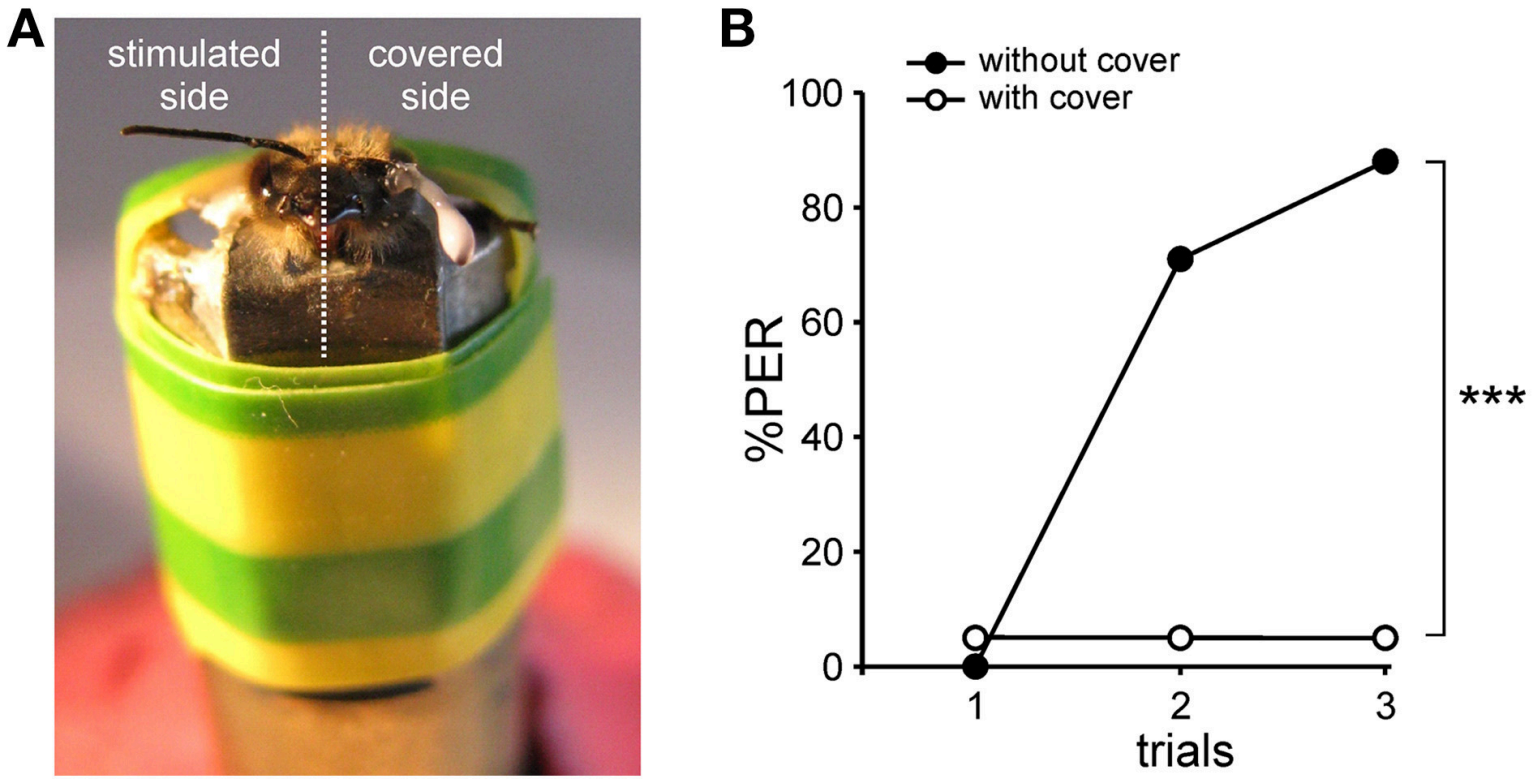
**Antenna cover efficiency. (A)** Photograph of a harnessed honey bee prepared for unilateral PER conditioning, with one antenna covered with latex.**(B)**Test of cover efficiency. Conditioning of the proboscis extension response (PER) in one group of bees with both antennae covered (*n* = 20) and in another group without any cover (*n* = 17). The percentage of PER increases in the course of training for the group without cover, whereas it remains almost null for the group with both antennae covered (^***^p < 0.001, Mann-Whitney test).

Worker bees were collected in the morning from the entrance of outdoor hives. To facilitate handling and mounting, bees were anesthetized on crushed ice for 5 min. They were then placed into individual metal tubes, taking care to leave their antennae, mandibles, and proboscis free. Two adhesive strips were placed behind the head and the abdomen. The bees were then fed with 5 μL sugar solution (50% w/w) to homogenize their satiety state. The lesions of the m-ALT being made unilaterally (to increase the success rate of this method), the appetitive olfactory conditioning also had to be performed unilaterally (Sandoz et al., 2002; Letzkus et al., 2006). For this reason, the antenna contralateral to the lesioned side was fixed with wax, and the flagellum was covered with 2-component silicone (Adisil rosé, Böhme & Schöps Dental, Goslar, Germany) to prevent odor detection on this side (Figure 2). The efficiency of the silicone for blocking olfactory input was checked in a group of bees with both antennae covered (see Results). After attaching the bee's head with wax, an opening was made with a razor blade between the compound eyes, and the detached piece of cuticle was preserved, so that it could be placed back after the brain lesion. To allow access to the brain and to perform the m-ALT lesion, glands, and trachea covering the brain were removed.

### Lesion and staining of m-ALT PNs

Lesion of the m-ALT was performed just prior to its entry at the level of the medial calyx of the MBs (Figure 1A). A glass electrode was coated with dye crystals (Tetramethylrhodamine dextran, 10000 kDa, Life technologies, France, mixed with 2% BSA) and was then inserted into the brain at the location of interest. As this dye only penetrates neurons when they are severed by the electrode, the lesioned neuronal tract can be later visualized under a confocal microscope (**Figure 5A**). The electrode was left at the same location for a few seconds, to allow the dye crystals to dissolve, thereby improving dye uptake by injured neurons. To control for the possible non-specific effect of the lesion, a group of bees received a similar lesion within the ipsilateral optic lobe, as done in previous studies (Erber et al., 1980; Hammer and Menzel, 1998; Farooqui et al., 2003). After the lesion, the head capsule of the bee was closed again with the preserved piece of cuticle to prevent the brain from drying out. Bees were then left in a calm, humid container for 2 h before PER conditioning.

### Olfactory conditioning of the PER

PER conditioning was performed in standard conditions (Bitterman et al., 1983; Matsumoto et al., 2012). A conditioning session consisted in five conditioning trials, in which an odor was associated with sucrose, separated by 10 min inter-trial intervals. The conditioned stimulus (CS) was the odorant 1-nonanol (C9-ol, Sigma, Deisenhofen, Germany). The presentation of the odor was performed manually at the bee's antennae, using a 20 mL syringe containing a 1 cm^2^ filter paper strip soaked with 5 μL of pure odor solution. The unconditioned stimulus (US) was a sugar solution (50% w/w) applied with a toothpick to the bees' uncovered antenna, and then to the proboscis. In the experiment with both antennae covered, both covered and uncovered bees received the US on the proboscis only. A conditioning trial lasted 30 s. One bee at a time was placed in the stimulation site in front of an air extractor and left for 15 s to accommodate to the experimental situation. Then, the CS (odor) was presented for 5 s and the US (sugar solution) was applied for the last 2 s of CS presentation. The interval between CS and US onsets was thus 3 s. The bee was left in the set up for 10 more seconds until the end of the trial. During conditioning, the responses (complete proboscis extension) to CS and US were recorded. Bees that did not respond to the US at any time during the experiment were excluded from the analysis as they were not considered motivated enough for the experiments.

### Generalization responses as a function of odor quality

To evaluate bees' generalization responses depending on odor quality, a session of unreinforced test trials was performed 10 min after conditioning. In these tests, the CS and five novel odorants, which differed from the CS in terms of their carbon chain length and/or functional group, were tested. The odorants were selected using the behavioral generalization matrix in Guerrieri et al. (2005), in order to obtain a regular descending gradient of generalization (see Results). Responses as a function of chain length were tested by using odors with the same functional group as the CS, but with different chain lengths: 1-octanol (C8-ol), 1-heptanol (C7-ol). Responses as a function of the functional group were tested by using C7 to C9 secondary ketones, 2-nonanone (C9-one), 2-octanone (C8-one), and 2-heptanone (C7-one). The six odorants were presented in a random order to the bees, except for the CS (1-nonanol, C9-ol), which was always presented last. The US was tested again at the end of the test session. As above, bees that did not respond to this US test were discarded from the analysis.

### A posteriori control of m-ALT lesions

To verify the quality of the m-ALT lesions, a post-behavior diagnostic was performed for all brains using confocal microscopy. After the generalization test session, the brains of all bees were removed and placed in paraformaldehyde (PFA) at 4% in PBS (Phosphate Buffer Saline, pH = 7.4) overnight for fixation. Subsequently, the brains were rinsed three times in PBS, and placed 3 h in a counter-staining solution, containing 0.2 U of phalloidin coupled with Alexa 488 (Molecular Probes, Eugene, OR, USA), 1% Triton X-100 and 500 μL PBS. Thereafter, the brains were rinsed three times in PBS, and then underwent a series of increasing alcohol baths for dehydration. Finally, the brains were clarified by placing them in methyl salicylate (Sigma-Aldrich, Deisenhofen, Germany) for at least 24 h. Brains were visualized using a confocal microscope (Leica SP5, Germany) equipped with an Argon laser, with a 10x objective. Tetramethylrhodamine was excited at 568 nm, and Alexa 488 at 488 nm. Data were acquired sequentially on both channels for each optical section (interval of 5 μm between sections). The data was then visualized and evaluated using ImageJ (National Institutes of Health, USA). Our selection criteria for establishing a positive diagnosis was clear visualization of either the tract (**Figure 5A**, left) or stained cell body clusters at the location of m-ALT PNs (**Figure 5A**, right). With these selection criteria, 85.5% of the bees showed an m-ALT lesion (*n* = 59 out of 69).

### Optical imaging of l-ALT PNs

*In vivo* calcium recordings of l-ALT PNs were performed under standard conditions, as detailed elsewhere (Carcaud et al., 2015). Shortly, bees were placed in Plexiglas recording chambers and the head capsule was opened revealing the brain. L-ALT PNs were stained with the calcium indicator Fura-2 dextran (potassium salt, 10000 kDa, in 2% BSA; Life technologies, France) using a glass electrode coated with dye crystals. The dye was inserted in the l-ALT axonal path, between the vertical lobe and the border of the optic lobe (OL), rostrally from the LH. After staining, the brain was immersed in standard bee saline solution and the bee was left in a moist and dark place for 3 h before imaging was performed. Measurements were obtained using a T.I.L.L. Photonics imaging set up (Martinsried, Germany), under an epifluorescent microscope (Olympus BX-51WI) with a 10x water-immersion objective (Olympus, UMPlanFL; NA 0.3). Fura-2 was alternatively excited with 340 nm and 380 nm monochromatic light (T.I.L.L. Polychrom IV). Each measurement consisted of 100 double frames, at a rate of 5 Hz (interval between double frames, 200 ms), with 4 × 4 binning on chip (pixel image size corresponded to 4.8 × 4.8μm). Integration time was 10–20 ms at 380 nm excitation and 40–80 ms at 340 nm excitation. Olfactory stimulation started at the 15^th^ frame until the 20^th^ frame, for 1 s. Each bee was subjected to three imaging sessions with 16 aliphatic odorants, belonging to four functional group types (primary and secondary alcohols, aldehydes and ketones) and carrying four different carbon chain lengths (6, 7, 8, and 9 carbons). In the present study, only data for the six odorants used in the behavioral tests (C9-ol, C8-ol, C7-ol, C9-one, C8-one, and C7-one) were analyzed. Odor stimuli were presented in a constant clean airstream at a distance of 2 cm from the bee's antennae. The interval between odor presentations was ~80 s. Imaging data were analyzed using custom-made software written in IDL 6.4 (Research Systems Inc., Boulder, CO, USA). The calcium response to each odor stimulation was calculated as the average of three frames during odor presentation (frames 17–19) minus the average of three frames just before stimulus delivery (frames 12–14). These responses are shown in a color code from dark blue to red in the glomerular activity maps. For analysis, a mask was precisely drawn around the AL of each bee and analysis was limited to the unmasked region. Evaluation of the similarity relationships between neural representations was assessed pixelwise, using an Euclidian metric (measure of dissimilarity) (Carcaud et al., 2015).

### Statistical analysis

Behavioral responses were scored in dichotomous form: a bee extends the proboscis (1) or not (0) at the presentation of the odorants, during conditioning or generalization tests. Cochran's Q test was used within group for comparing the responses of bees in the different acquisition trials or to the different odorants in the generalization tests. To compare acquisition success between groups, Mann-Whitney U tests were performed on the sum of each bee's responses to the 5 conditioning trials. McNemar Chi^2^ tests were carried out to compare bees' responses to the CS at the 5^th^ conditioning trial and at the end of the test session. Lastly, Fisher's exact tests were performed to compare between groups the percentage of bees showing no response to the CS. Pearson correlation analyses were performed between generalization responses to the six tested odorants in different groups, or between generalization responses and inter-odor neural distances. The significance threshold for all analyzes was *p* < 0.05. All analyses were performed using STATISTICA 5.5 (Statsoft, Tulsa, USA).

## Results

Bees were subjected to a selective lesion of the m-ALT followed by an appetitive olfactory conditioning of the PER and a test of their generalization responses to odorants varying in chemical quality. Bees with a lesion in the OL, a structure that is not involved in olfactory processing, and untreated bees without any damage, were used as controls and subjected to the same olfactory conditioning and generalization tests.

### Antenna cover efficiency

The m-ALT lesions were performed unilaterally to optimize the proportion of successfully lesioned animals. Thus, appetitive olfactory conditioning was also carried out unilaterally by delivering the conditioned odor on the antenna corresponding to the lesioned side. The contralateral antenna was covered with two-component silicon to prevent odor detection on this side (Figure 2A). The efficiency of the silicon cover for blocking olfactory input was first tested on bees with both antennae covered (Figure 2B). These bees received three 1-nonanol (CS)—sucrose (US) associations, yet none learned to respond to the CS during training (one bee responded spontaneously to the odor during the three trials). By contrast, a control group with both antennae uncovered learned very efficiently the odor-sucrose association (Cochran Q test, *Q* = 25.2, 2 df, *p* < 0.001) and reached 88% conditioned responses in the third trial (Figure 2B). The difference between the two groups was highly significant (Mann-Whitney test, Z_adj_ = 4.61, *p* < 0.001), thus showing that the silicon cover prevents olfactory detection and therefore learning. Unilaterally covered bees can thus only learn using their uncovered side.

### Unilateral conditioning of intact bees

**Figure 3:**
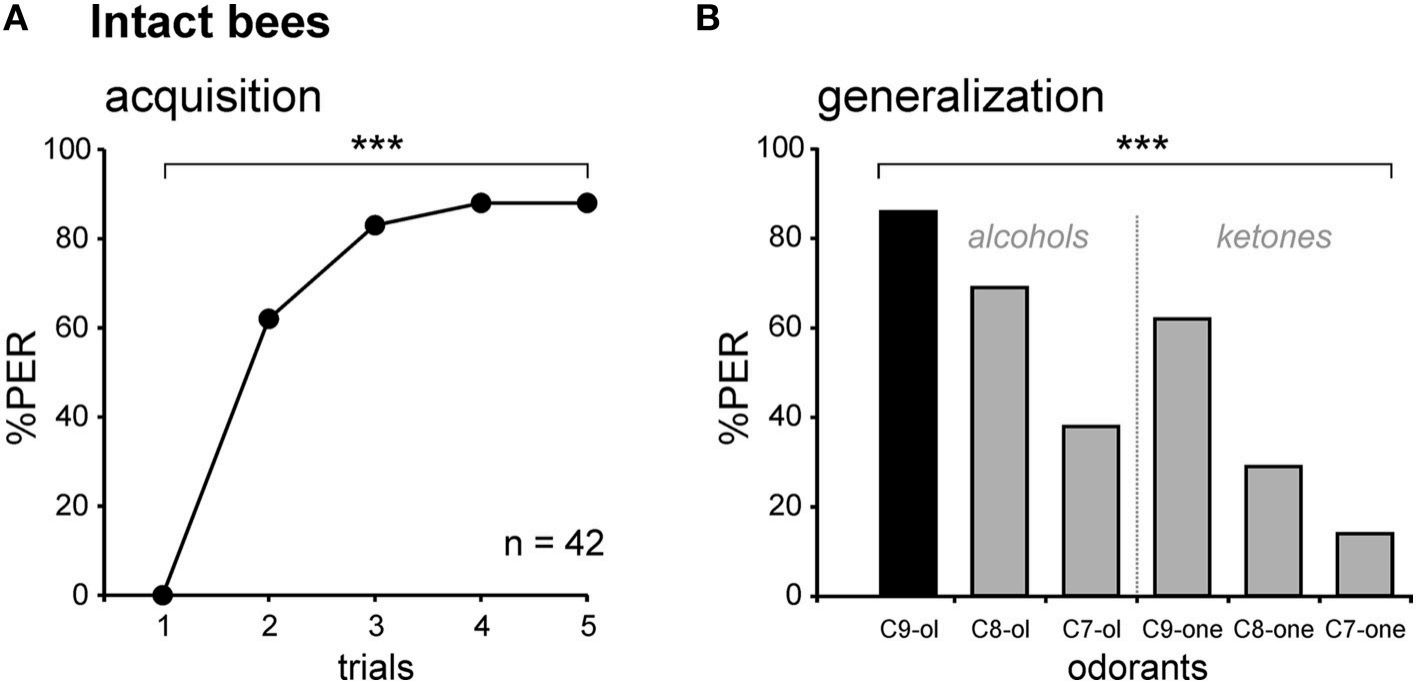
**Associative olfactory conditioning and generalization tests on intact honey bees. (A)** Intact bees learn to associate the odorant CS (1-nonanol, C9-ol) with the sucrose US, as shown by the increase in the PER percentage along trials (*n* = 42; ^***^p < 0.001, Cochran's Q test). **(B)** Generalization tests after PER conditioning, using 5 new odorants which differed from the CS by their chain length (C8-ol and C7-ol), by their functional group (C9-one) or both (C8-one, C7-one). Intact bees respond differentially to the tested odorants (^***^p < 0.001, Cochran's Q test), depending on their chemical properties, and show no extinction. Abbreviations: C8-ol, 1-octanol; C7-ol, 1-heptanol; C9-one, 2-nonanone; C8-one, 2-octanone; C7-one, 2-heptanone.

We first measured olfactory learning and generalization performances in intact bees (i.e., without any lesion). All bees exhibited unconditioned PER to sugar solution (US, *n* = 42). When subjected to olfactory PER conditioning, the percentage of bees showing conditioned PER to the CS (1-nonanol, C9-ol) increased in the course of training (Cochran Q test, *Q* = 116.9, 4 df, *p* < 0.001), showing that they learned to associate this odor with sucrose (Figure 3A). At the end of the training (5^th^ trial), 88% of the bees responded to the CS. After training, five new odorants and the CS were presented to the bees, without any reinforcement (generalization test). The novel odors differed from the CS in their chain length (C8-ol and C7-ol), their functional group (C9-one) or in both (C8-one, C7-one). Bees responded differently to the tested odorants (Cochran Q test, *Q* = 81.6, 5 df, *p* < 0.001; Figure 3B). They showed generally more PER to primary alcohols, the functional group of the CS, than to ketones. They also showed more PER to long-chain molecules, especially C9, the chain length of the CS, than to shorter chain lengths. These generalization tests potentially pose the problem of extinction of the CS-US association, because odorants are delivered without reward in these tests (Bitterman et al., 1983; Sandoz and Pham-Delègue, 2004). We tested this possibility by comparing the bees' responses to the CS at the end of conditioning (5^th^ trial) and at the end of the generalization tests (last test trial). We found that bees responded at the same level to both CS presentations (McNemar test, Chi^2^ = 0, NS), indicating that the presentation of five non-reinforced novel odorants in the tests did not induce any significant extinction.

### Unilateral conditioning of control bees with lesions in the optic lobes

**Figure 4:**
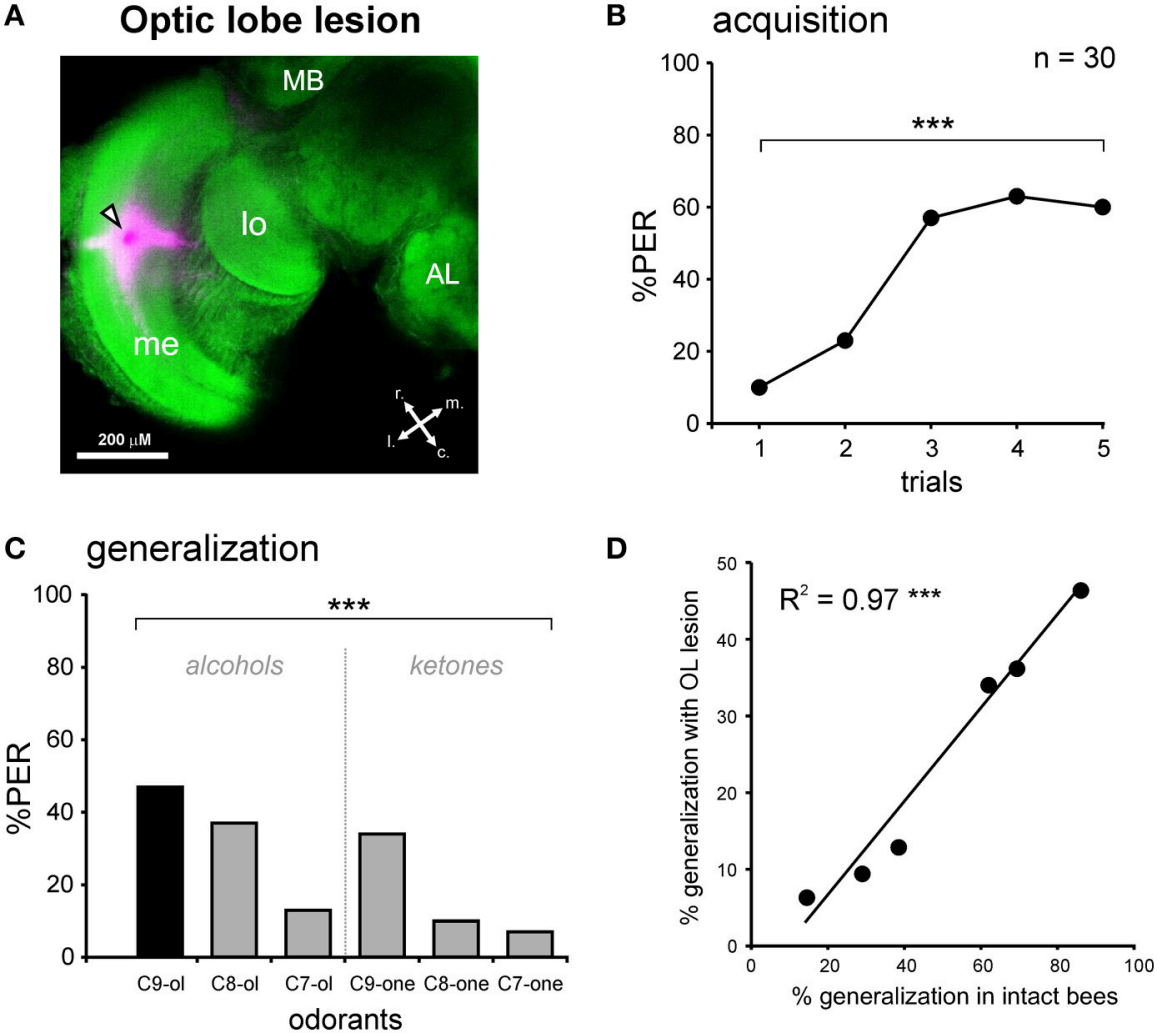
**PER conditioning and generalization tests after an optic lobe lesion. (A)** Staining during the OL lesion using tetramethylrhodamine and counter-staining with Alexa488 coupled to phalloidin. Abbreviations: MB, mushroom body; me, medulla; lo, lobula; r, rostral; c, caudal; l, lateral; m, medial. The black and white arrowhead points to the lesion site. **(B)** PER conditioning in bees with an OL lesion. OL-lesioned bees manage to associate the odor CS (1-nonanol, C9-ol) with the sucrose reward (US), as shown by the increase in the PER percentage along trials (*n* = 30; ^***^p < 0.001, Cochran's Q test). **(C)** Generalization tests after PER conditioning, using five new odorants differing from the CS by their chain length and/or by their functional group. Bees with an OL lesion respond differentially to the tested odorants (^***^p < 0.001, Cochran's Q test). **(D)** Highly significant correlation between the responses of bees with an OL lesion and the responses of intact bees, during generalization tests (^***^p < 0.001, *R*^2^ = 0.97).

We performed the same conditioning and generalization tests using bees injured in the optic lobes (Figure 4A). This experiment allowed studying the effect of the operation and of brain injury *per se* without involving the m-ALT. It thus represents a good sham control for the m-ALT lesions. The surgical procedure resulted in 13.1% of the bees that did not show an unconditioned PER to the sucrose solution (US). This proportion was significantly higher than that of untreated bees which all responded to the US (Fisher's exact test, *p* < 0.001). Only bees that responded to the US were further used in the unilateral olfactory PER conditioning. These bees were also capable of associating the odor CS with the sucrose US and increased their responses to this odorant along trials (*n* = 30, Cochran's Q test, *Q* = 42.6, 4 df, *p* < 0.001; Figure 4B). At the end of conditioning (5^th^ trial), 60% of the bees responded to the CS. Compared to intact bees, the operation induced a slight but significant learning deficit (**Figure 6**, Mann-Whitney test, Z_adj_ = −3.04, *p* < 0.01). In the generalization tests, bees responded to the CS at the same level as at the end of training (5^th^ training trial, McNemar test, Chi^2^ = 1.13, NS). Bees responded however differently to the six tested odorants (Cochran Q test, *Q* = 30.5, 5 df, *p* < 0.001). Like intact bees, OL-lesioned bees responded more to primary alcohols than to ketones, and more to odorants with a long carbon chain than with a shorter one (Figure 4C). Thus, bees with an OL lesion showed a generalization response that was almost identical to that of control bees (Figure 4D, Pearson correlation, *R*^2^ = 0.97, 4 df, *p* < 0.001). We can thus conclude that the surgical operation and the brain damage induced outside of the olfactory pathway slightly affect the proportion of learning bees, but do not affect the generalization response.

### Unilateral conditioning of bees with an m-ALT lesion

**Figure 5:**
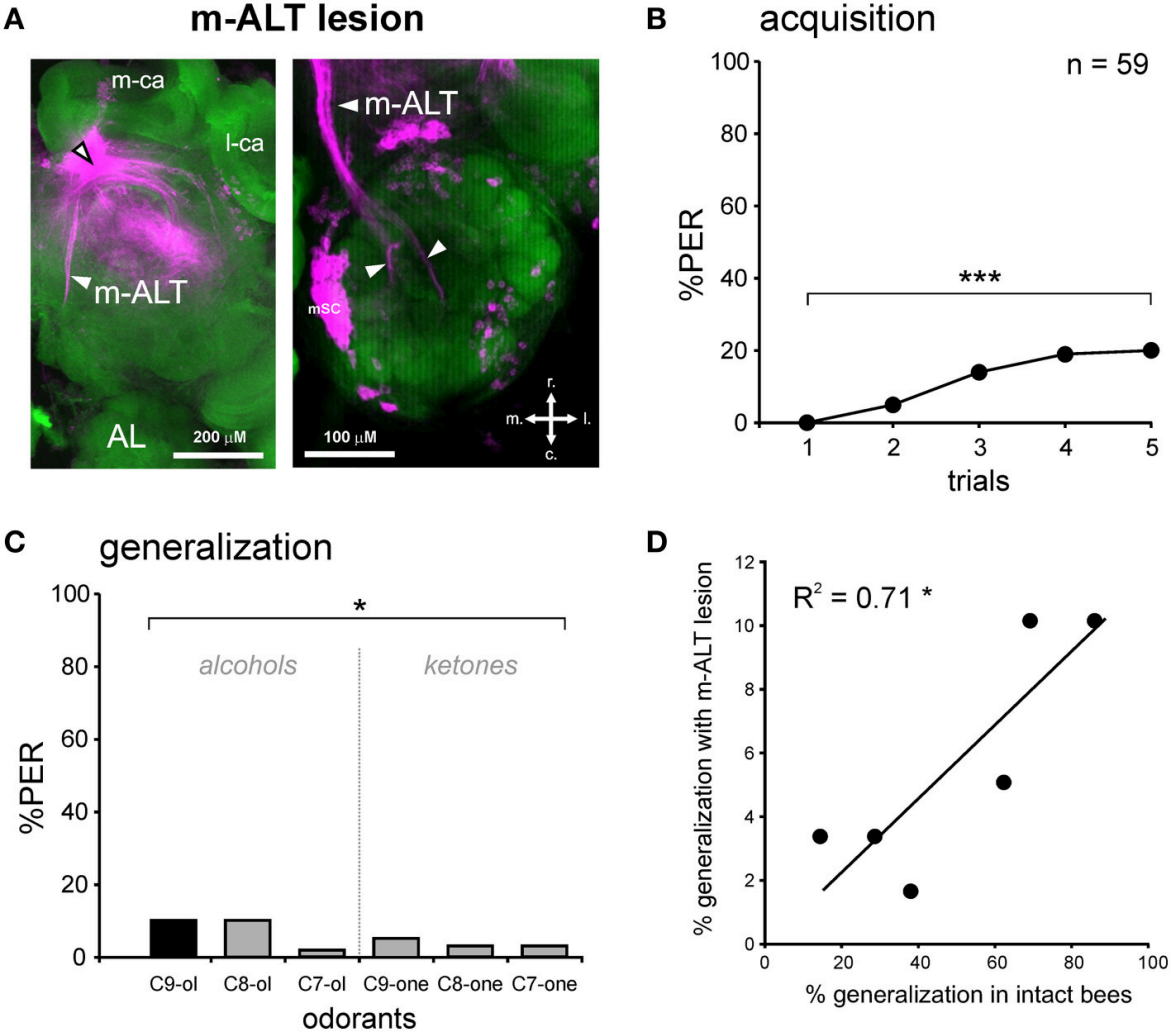
**PER conditioning and generalization tests after an m-ALT lesion. (A)** Staining with tetramethylrhodamine induced by the m-ALT lesion and Alexa488 coupled to phalloidin as counter-staining. The black and white arrowhead points to the lesion site. A stained m-ALT tract (left) or the stained somata clusters of m-ALT PNs (mSC, right) were used as indicators for a successful lesion. Abbreviations: r, rostral; c, caudal; l, lateral; m, medial. **(B)** PER conditioning in bees with an m-ALT lesion. Only a few m-ALT lesioned bees managed to associate the odor CS (1-nonanol, C9-ol) with the sucrose reward (US), as shown by the weak increase in PER percentage with trials (*n* = 59; ^***^p < 0.001, Cochran's Q test). **(C)** Generalization tests after PER conditioning, using five new odorants, shows that bees with an m-ALT lesion respond differentially to the different odorants (^*^p < 0.05, Cochran's Q test). **(D)** Significant correlation between the responses of bees with an m-ALT lesion and intact bees during generalization tests (^*^p < 0.05, *R*^2^ = 0.71).

**Figure 6:**
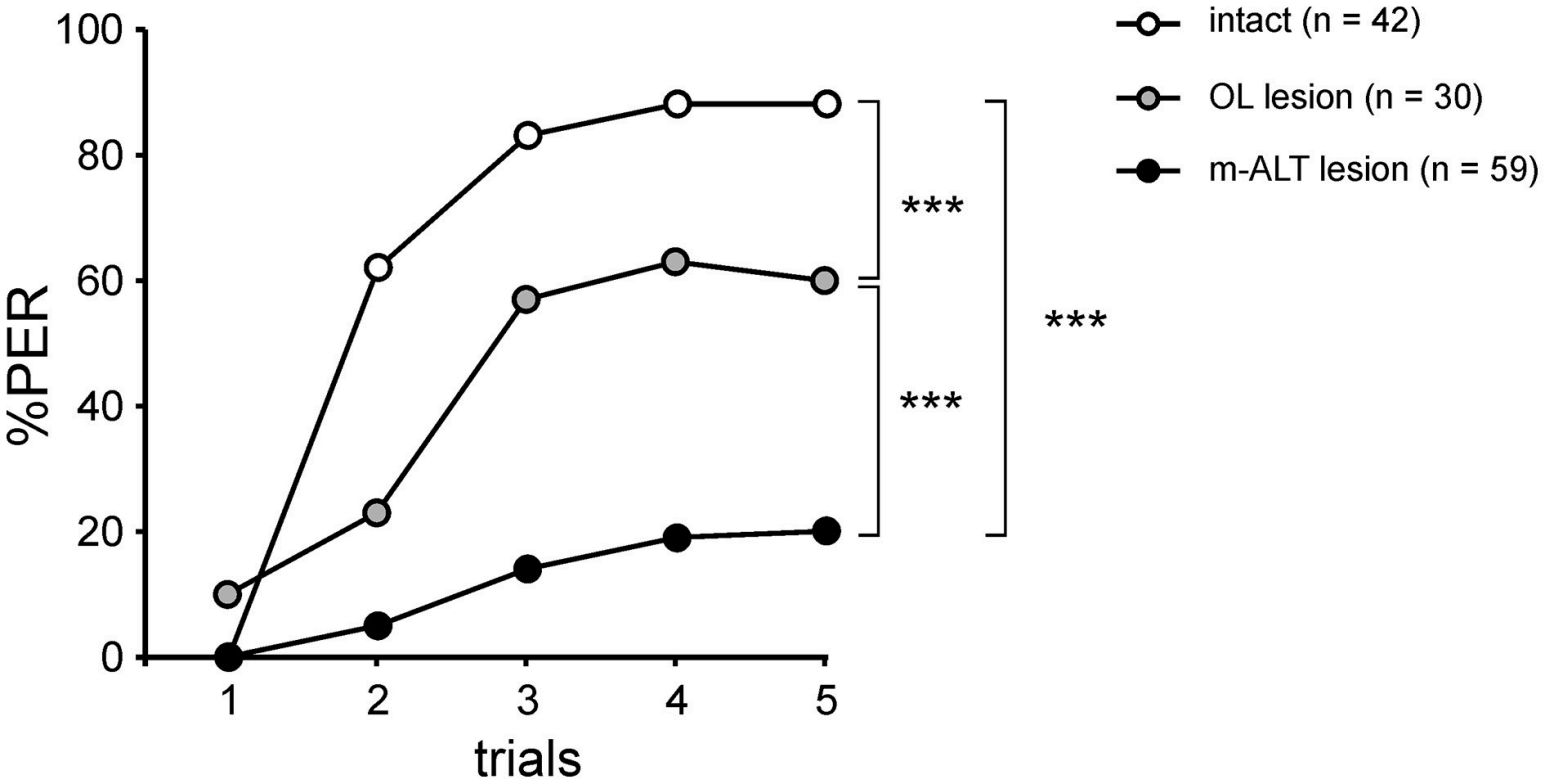
**PER conditioning performances in the three experimental groups**. Acquisition performances are significantly lower in bees with an OL lesion than in intact bees, showing an effect of surgery (^***^p < 0.001, Mann-Whitney test). PER conditioning performances are much weaker in bees with an m-ALT lesion than in bees with an OL lesion (^***^p < 0.001, Mann-Whitney test), demonstrating a specific effect induced by the m-ALT PN lesion.

In the group with a unilateral m-ALT lesion (Figure 5A), 14.5% of the bees did not respond to the US, a proportion that was similar to that observed in bees with an OL lesion (Fisher's exact test, NS). Thus, the fact that the lesion was applied on the m-ALT did not reduce the bees' responsiveness to the sucrose US. As in the previous experiments, only bees that responded to the US were kept for PER conditioning. These bees showed only a slight increase in their PER to the CS during trials, which was nevertheless significant (*n* = 59, Cochran Q test, *Q* = 28.1, 4 df, *p* < 0.001). At the end of training (5^th^ trial), only 20.3% of the bees responded to the CS, a significantly lower proportion than in bees with an OL lesion (Figure 6, Mann-Whitney test, Z_adj_ = 4.42, *p* < 0.001). This result shows that an m-ALT lesion induces a strong acquisition deficit, which is not induced by general brain damage but by the lesion of the PN tract. In the generalization tests, m-ALT lesioned bees showed a slight, but non-significant reduction in performances to the CS (10.2%) compared to the end of training (5^th^ training trial, McNemar test, Chi^2^ = 3.13, NS). Despite the low level of conditioned responses, bees showed differential generalization responses to the 6 tested odorants (Figure 5C, Cochran Q test, *Q* = 11.7, 5 df, *p* < 0.05). Like intact and OL-lesioned bees, bees with an m-ALT lesion responded mostly to novel odorants with a similar chain length (C9-one) or a similar functional group (C8-ol) as the CS. Accordingly, we found a significant correlation between the generalization responses of m-ALT lesioned bees and those of both intact (Figure 5D, *R*^2^ = 0.71, 4 df, *p* < 0.05) and OL-lesioned bees (*R*^2^ = 0.76, 4 df, *p* < 0.05, not shown). This analysis was also performed by using only bees that still responded to the CS at the end of the generalization phase (Supplementary Figure 1). This analysis confirmed a significant correlation of the generalization responses of m-ALT lesioned bees (*n* = 6 bees) with those of intact bees (*n* = 36 bees, *R*^2^ = 0.75, 4 df, *p* < 0.05) as well as those of OL-lesioned bees (*n* = 14 bees, *R*^2^ = 0.79, 4 df, *p* < 0.05). This experiment thus suggests that m-ALT PNs are necessary for efficient acquisition in olfactory PER conditioning but that their lesion does not hinder olfactory generalization in learners.

### Comparison of generalization responses with optical imaging data

**Figure 7:**
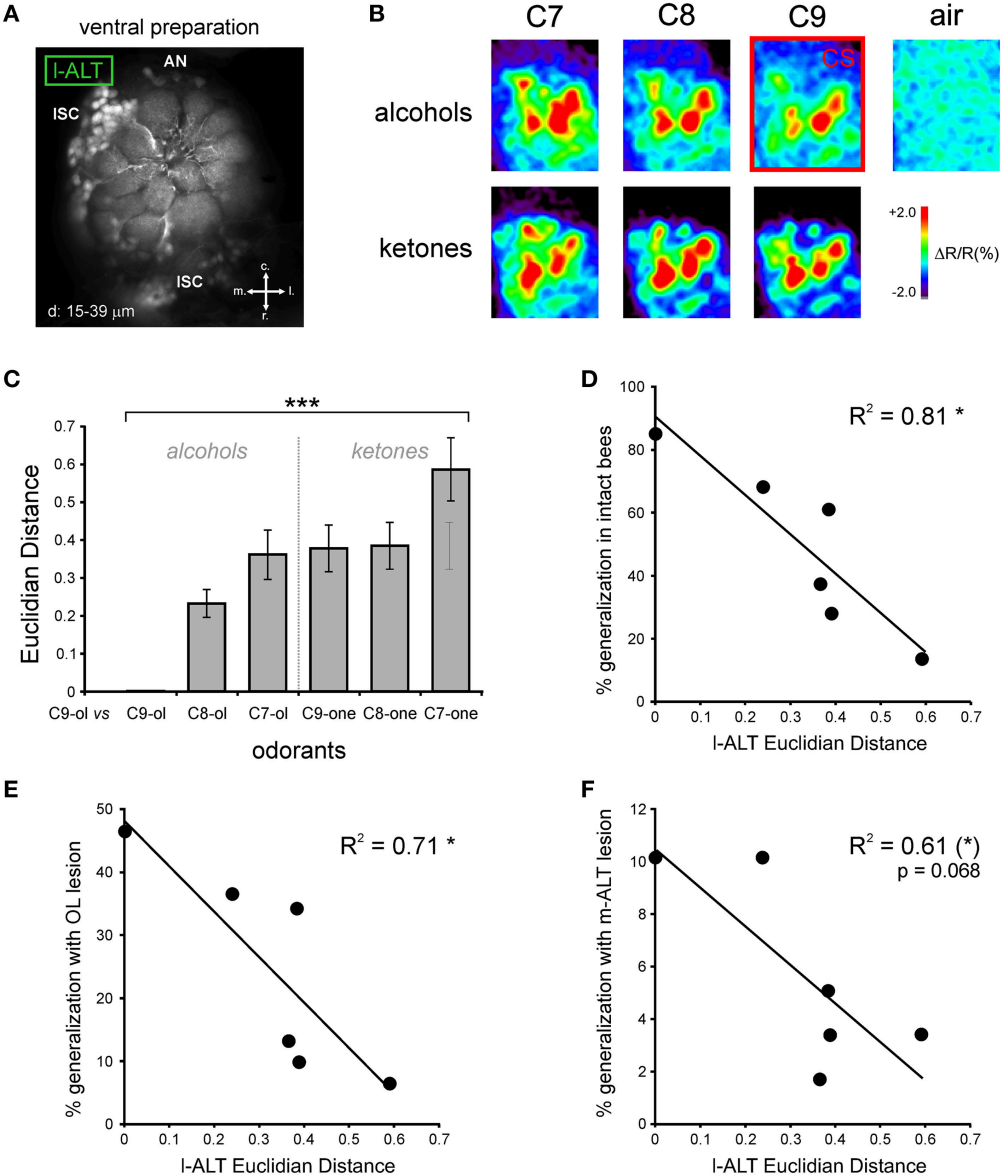
**Comparison of neural coding in l-ALT neurons with behavioral performances. (A)** Retrograde staining of l-ALT PNs innervating ventral AL glomeruli (adapted from Carcaud et al., 2015). Z-projection of optical slices performed at the indicated depths (d). Abbreviations: r, rostral; c, caudal; l, lateral; m, medial; lSC, l-ALT PN somata cluster; AN, antennal nerve. **(B)** Odor-induced calcium signals in l-ALT PNs in the AL to the six different odorants, differing in their functional group (alcohol and ketone) and their carbon chain length (7, 8, and 9 carbons) and to the air control. The CS in the behavioral experiment (1-nonanol; C9-ol) is framed in red. **(C)** Euclidian distances (dissimilarity measure between activity maps) calculated between the CS (C9-ol) and the five new tested odorants are significantly different (^***^p < 0.001, repeated measure ANOVA). **(D–F)** Significant and similar correlations between l-ALT Euclidian distances and the responses in the generalization tests of: **(D)** intact bees (*R*^2^ = 0.81, ^*^p < 0.05); **(E)** bees with an OL lesion (*R*^2^ = 0.71, ^*^p < 0.05); and **(F)** bees with m-ALT lesion (*R*^2^ = 0.61, (^*^)*p* = 0.068).

The previous result suggests that after lesion of m-ALT PNs, a low proportion of bees still display a decreasing response gradient to increasingly chemically different odorants in generalization tests. One possibility to explain this finding would be that generalization responses in these individuals would be possible thanks to the other unilateral PN pathway, the l-ALT (Figures 1, 7A). We thus asked whether the generalization gradient shown by m-ALT lesioned bees could be explained by neural activity from l-ALT PNs only. Using *in vivo* calcium imaging of l-ALT PNs in the AL, we recorded glomerular activity patterns for the six odorants used in the present study (Figures 7A,B). Each odorant induced activity in a specific combination of AL glomeruli, which differed between odorants (Figure 7B). These maps were used to measure neural similarity relationships among odorants in this olfactory subsystem. Neural similarity between the glomerular response maps of the C9-ol (corresponding to the CS in the behavioral experiment) and the other five odorants are presented in Figure 7C as pixelwise Euclidian distances, with longer Euclidian distances corresponding to more dissimilar neural maps (*n* = 10 bees). As observed previously (Sachse et al., 1999; Szyszka et al., 2011; Carcaud et al., 2012), similarity among neural response maps was higher (i.e., shorter Euclidian distances) when odorants had the same functional group as the CS (C8-ol, C7-ol) or had a similar carbon chain length (C9-one). We then represented the behavioral data of the three groups of bees (intact, OL-lesion, m-ALT lesion) as a function of the neural distance measured between the CS and each tested odorant (Figures 7D–F). In all three groups, the more similar the l-ALT maps between the tested odorant and the CS were, the more the bees generalized in the behavioral tests. The correlation between behavioral generalization and the neural distances was significant for intact bees (Figure 7D, *R*^2^ = 0.81, 4 df, *p* < 0.05), for OL-lesioned bees (Figure 7E, *R*^2^ = 0.71, 4 df, *p* < 0.05), and near-significant for m-ALT lesioned bees (m-ALT lesioned bees: Figure 7F, *R*^2^ = 0.61, 4 df, *p* = 0.068). In this last group, the prediction appeared less accurate, which may be related to the discrete nature of the behavioral data and the low number of individuals showing responses during generalization tests in this group (see above).

## Discussion

We performed unilateral lesions of the m-ALT PN tract in honey bees, and showed that this procedure strongly affects the acquisition of an odor-sucrose association. However, in the few individuals that managed to learn the association, olfactory generalization was preserved, as these bees responded generally more to an odorant when its chain length or functional group was the same as that of the learned odorant. The generalization responses in m-ALT lesioned bees correlated with those of both intact and optic-lobe lesioned bees. In addition, they could be to some extent predicted by a neural similarity measure based on optical imaging responses of l-ALT PNs.

### Non-specific lesion effects

We used glass electrodes to produce local lesions of PNs of the m–ALT tract in the bee brain. Such procedure is routinely used in neuroanatomical and optophysiological studies in bees, in which normal neural responses can be recorded at the level of the AL, thus suggesting that olfactory processing is functional prior to the m-ALT lesion stage (Sachse and Galizia, 2002; Szyszka et al., 2005; Carcaud et al., 2015; Peele et al., 2006). However, because of the lesion, the neural message normally conveyed to higher-order brain centers by these neurons is not transmitted anymore. As a control for the m-ALT lesions, we used a lesion of the same size, but within the OL, i.e., a brain region totally devoid of olfactory processes. Bees presenting this lesion showed a slight decrease in their responses to the sucrose US and a significant reduction in their acquisition success. These results can be interpreted as a general detrimental effect of the lesion on the bees' physiological state and/or appetitive motivation, due to the invasive nature of the lesion. Studies using invasive procedures (e.g., *in vivo* electrophysiology) and/or brain injections also induce reduced learning performances compared to routine PER experiments with intact animals, but in most cases these reductions could not be unambiguously attributed to the preparation because these studies did not contain an intact control group (e.g., Faber et al., 1999; Malun et al., 2002; Rath et al., 2011). In the case of selective-lesion studies, such a group appears decisive for correct data interpretation. Our conclusions on the effects of m-ALT lesions are primarily based on the comparison between this group and the control group presenting an OL lesion. Nonetheless, we need to mention two possible limitations of our approach. First, although we obtained a clear diagnosis for each bee showing that the m-ALT tract had been lesioned, we cannot ensure that in all animals all m-ALT PNs were cut (see below). Second, on its way to the m-ALT PN tract, the electrode could have also affected other local protocerebral neurons, including some MB neurons. Although these local neurons may not be directly related to the olfactory pathway, we cannot totally exclude the possibility of collateral effects on protocerebral neurons participating in the observed acquisition deficit.

### A role for PNs in olfactory acquisition

Previous neurophysiological studies showed that l-ALT and m-ALT neurons respond to a mostly redundant array of general odorants (i.e., non-pheromonal odorants), albeit with somewhat different spatiotemporal characteristics (Müller et al., 2002; Krofczik et al., 2009; Carcaud et al., 2012; Galizia et al., 2012; Brill et al., 2013). This observation suggested that both neural tracts could be functionally redundant for the learning of these odorants. In particular, optical imaging recordings showed clearly that both l-ALT and m-ALT subsystems respond to the odorant 1-nonanol, the CS we used to train the bees (Carcaud et al., 2012, 2015). Therefore, if both subsystems were totally redundant, bees should be perfectly able to learn to associate 1-nonanol with sucrose even in the absence of a functional m-ALT tract. This was not the case as bees with an m-ALT lesion showed a strong decrement of acquisition and retrieval performances compared to bees with an OL lesion.

This result contradicts the common idea that normal olfactory function within the antennal lobe alone is sufficient for olfactory acquisition. This idea stems from the results of several studies. Erber et al. (1980) were the first to suggest a role for the AL in appetitive olfactory learning by showing that local cooling of the AL in the first 3 min after a single-trial conditioning strongly reduces bees' conditioned responses. Later, this role was confirmed by Hammer and Menzel (1998), who showed that injection in the AL of octopamine, the neurotransmitter mediating the reinforcing properties of the sucrose US, is sufficient for inducing significant acquisition if it follows immediately an odor presentation. In the same line, Farooqui et al. (2003) confirmed that blocking OA neurotransmission in the AL also blocks acquisition. Together, these results indicate that an olfactory memory supporting normal acquisition performance is established through association of the odor CS and OA-mediated US information in the AL. Other studies repeatedly showed appetitive learning-induced plasticity both in the structure and activity of AL networks (Faber et al., 1999; Fernandez et al., 2009; Hourcade et al., 2009; Rath et al., 2011; Arenas et al., 2012). However, these data do not give any insights into the role of connecting processes between AL and MBs. Our results suggest that after the formation of a CS-US association in the AL, associative plasticity would be transmitted via PNs to the MB calyx for further acquisition and memory consolidation. Such transfer processes are also found in other memory systems, for instance between the hippocampus and the cortex (Takashima et al., 2006; Durrant and Lewis, 2009; Preston and Eichenbaum, 2013) or between the cerebellum and the vestibular nuclei (Shutoh et al., 2006; Okamoto et al., 2011). The drop in acquisition after an m-ALT lesion could either indicate that the m-ALT tract alone is involved in such transfer, or that concomitant activity from both m-ALT and l-ALT neurons is necessary for this task. At this time, it is difficult to decide between these two hypotheses, because up to now no study could perform a perfectly specific l-ALT lesion. However, one previous study has provided interesting clues. As a control for an optical imaging experiment, Peele et al. (2006) applied an l-ALT lesion between the LH and the MB calyces in one hemisphere of the bee brain. The authors observed a similar effect as in the present study: unilaterally lesioned bees conditioned with a bilateral CS did not respond to this CS when it was presented on the lesioned side (Peele et al., 2006). If the observed effect was due to the l-ALT lesion, this data suggest that the l-ALT is also necessary for normal olfactory learning. Yet, in this study it is unclear if the applied lesion also severed the m-ALT, which is also found at this location. Therefore, if the observed effect was rather due to the m-ALT lesion, it would suggest that the LH would be the target of the plastic message carried by m-ALT neurons. Only further work with specific lesions of the l-ALT or m-ALT at different locations in the brain may help clarify this point. In any case, our results together with the study by Peele et al. (2006) identify a prominent role of PNs in olfactory learning performance.

### Hypotheses about possible mechanisms

The PNs of the honey bee are well-known for their associative learning-related plasticity, as shown repeatedly for l-ALT neurons (Fernandez et al., 2009; Rath et al., 2011; Chen et al., 2015). After olfactory conditioning, the odor representation of a learned odorant is modified in such a way that some glomeruli (some PNs) see their activity increased, while others see their activity decreased (see also Denker et al., 2010). The net result is a change in the PN representation of the learned odorant, possibly facilitating the detection of learned odors and their discrimination from other environmental odorants (Rath et al., 2011; Sandoz, 2011). Therefore, the plasticity observed in l-ALT neurons has mostly been associated with an improved detection of the CS, but not for subtending the CS-US association. To this day, we have very little data about a possible plasticity of m-ALT neurons. How would such PNs' activity change with learning in such a way that they could inform downstream neurons that an odorant has been learned? One may speculate that the neural activity of PNs responding to a learned odor contains a particular signature, for instance in the form of increased coincidence among these PNs. A recent study used extracellular recordings to measure neural activity simultaneously from l-ALT and m-ALT PNs, thereby quantifying coincident activity between neurons both within each PN tracts and between tracts (Brill et al., 2015). The study demonstrated that coincidence probability is significantly above random level among neurons of each tract, as well as between neurons of both tracts. Coincidence levels were especially high within the m-ALT. The authors proposed that coincident activity may play a role in olfactory processing. Considering the data of our study, one may hypothesize that such coincident activity could increase specifically for a learned odorant, either between PNs of the m-ALT or between PNs of the m-ALT and l-ALT. It would therefore be important now to use the methodology developed by Brill et al. (2013, 2015) to measure coincident activity to odorants between PNs of the l-ALT and m-ALT before and after appetitive conditioning. Given the indicated stability of such extracellular recordings, this type of experiment is feasible.

### Coincidence at the KC level and MB extrinsic neurons

Increased coincidence between PNs could be read out at the calyx level by the intrinsic MB neurons, the Kenyon cells. In the bee, the ~800 PNs diverge onto a major proportion of the 170,000 KCs of each MB (i.e., onto olfactory KCs). Each PN contacts many KCs and each KC receives input from many PNs. KCs do not show any spontaneous activity, and respond to very few odorants, giving rise to a highly sparse representation at the MB level (Perez-Orive et al., 2002; Szyszka et al., 2005; Turner et al., 2008; Honegger et al., 2011). The low synaptic strength existing between PNs and KCs implies that coherent input from many PNs at the same time is needed for exciting a KC (Perez-Orive et al., 2002). Therefore, a higher coincidence between PNs responding to the learned odorant may increase the probability of coincident activation of KCs by this odorant. The importance of this coincident activation of KCs for associative changes in MB extrinsic neurons has been recently demonstrated in locusts, through modification of spike-timing dependent plasticity rules at these synapses (Cassenaer and Laurent, 2012). Eventually, these processes could participate in the drastic changes observed in MB extrinsic neurons after olfactory learning (Okada et al., 2007; Strube-Bloss et al., 2011; Owald et al., 2015). The m-ALT lesion performed in our work would drastically reduce the transmission of coincidence input to the MBs, resulting in lower coincidence among KCs and lower performance levels.

### Conserved generalization performances after m-ALT lesion

Although acquisition was strongly reduced by the m-ALT lesion, we observed a clear generalization gradient in the few individuals that learned the CS-US association. M-ALT injured bees responded to each tested odorant according to its similarity with the CS, both in terms of chain length and of functional group. In addition, generalization in these bees was similar to that displayed by intact and optic-lobe lesioned bees. Two hypotheses could explain this finding. First, in these individuals, the olfactory message from the other, intact tract of uniglomerular PNs (l-ALT PNs) could have been sufficient to support both acquisition and adequate generalization. This idea is substantiated by the fact that the generalization responses of m-ALT lesioned bees showed a clear tendency to correlate (*p* = 0.068) with neural similarity measures obtained from optical imaging recordings of l-ALT PNs. Within the framework of the coincidence hypothesis developed above, one could propose that coincidence among l-ALT PNs (Brill et al., 2015) increased in response to the CS and that this would be sufficient to support learning performance in these bees. During the generalization tests, the olfactory maps from l-ALT PNs alone, inducing activity from partially overlapping groups of KCs, would allow the bees to show a gradual decrement of conditioned responses to increasingly chemically-different odorants. An alternative hypothesis that cannot be excluded revolves around the possibility that m-ALT lesions were only partial in some animals, despite their important size. It is therefore possible that in the small proportion of m-ALT lesioned individuals that learned successfully, some PNs responding to 1-nonanol were intact and supported acquisition. This hypothesis is, however, less robust when applied to the generalization results. It requires indeed that in these same individuals enough PNs were also left intact for supporting a normal generalization to the five other odorants. It seems, therefore, more parsimonious to suggest that the observed generalization gradient was mediated by neurons from the l-ALT tract, which was whole in these bees. Applying the m-ALT lesion between acquisition and generalization tests could allow a future experimental test of this hypothesis.

## Conclusion

In recent years, intense discussions have arisen about whether the two PN pathways of the honey bee represent two segregated information streams or if they serve a parallel processing function. The general view emerging from a series of different studies (for review, Galizia and Rössler, 2010; Sandoz, 2011; Nawrot, 2012; Rössler and Brill, 2013) is that both pathways mostly respond to a broad spectrum of odorants, albeit with somewhat different spatiotemporal characteristics (Müller et al., 2002; Krofczik et al., 2009; Carcaud et al., 2012; Galizia et al., 2012; Brill et al., 2013). Our results show that, for olfactory learning, both tracts are not redundant channels. The m-ALT seems to be critical for successful appetitive olfactory learning. We do not know whether both m-ALT and l-ALT are necessary for this task, or if the l-ALT is dispensable. The lesion strategy employed in this work for the first time could be instrumental for progressing on this question. It will be important to refine its application, in terms of the size and precision of the lesion, especially if we want to perform specific l-ALT lesions. One interesting possibility toward this goal will be the use of 2-photon laser mediated microdissection (Strutz et al., 2014).

## Author contributions

JC, MG, and JCS designed the experiments. JC performed all experiments. JC and JCS analyzed data and drafted the MS. All authors participated in the final version of the MS.

### Conflict of interest statement

The authors declare that the research was conducted in the absence of any commercial or financial relationships that could be construed as a potential conflict of interest.

## Supplementary material

Supplemental Figure 1 **PER conditioning and generalization performances in m-ALT lesioned bees, after selection of bees responding to the CS at the end of the generalization phase. (A)** Acquisition performances (*n* = 6 bees). **(B)** Responses in the generalization tests. **(C,D)** Significant correlation between the responses of these m-ALT lesioned bees (*n* = 6) during generalization tests with those of intact bees (**C**, *n* = 36 bees, ^*^*p* < 0.05, *R*^2^ = 0.75) and OL-lesioned bees (**D**, *n* = 14 bees, ^*^*p* < 0.05, *R*^2^ = 0.79).
