# Supplementary figures and images for: Parallel Olfactory Processing in the Honey Bee Brain: Odor Learning and Generalization under Selective Lesion of a Projection Neuron Tract
Source: Front Integr Neurosci. 2016 Jan 19;9:75. doi: 10.3389/fnint.2015.00075 (PMC4717326; doi:10.3389/fnint.2015.00075)

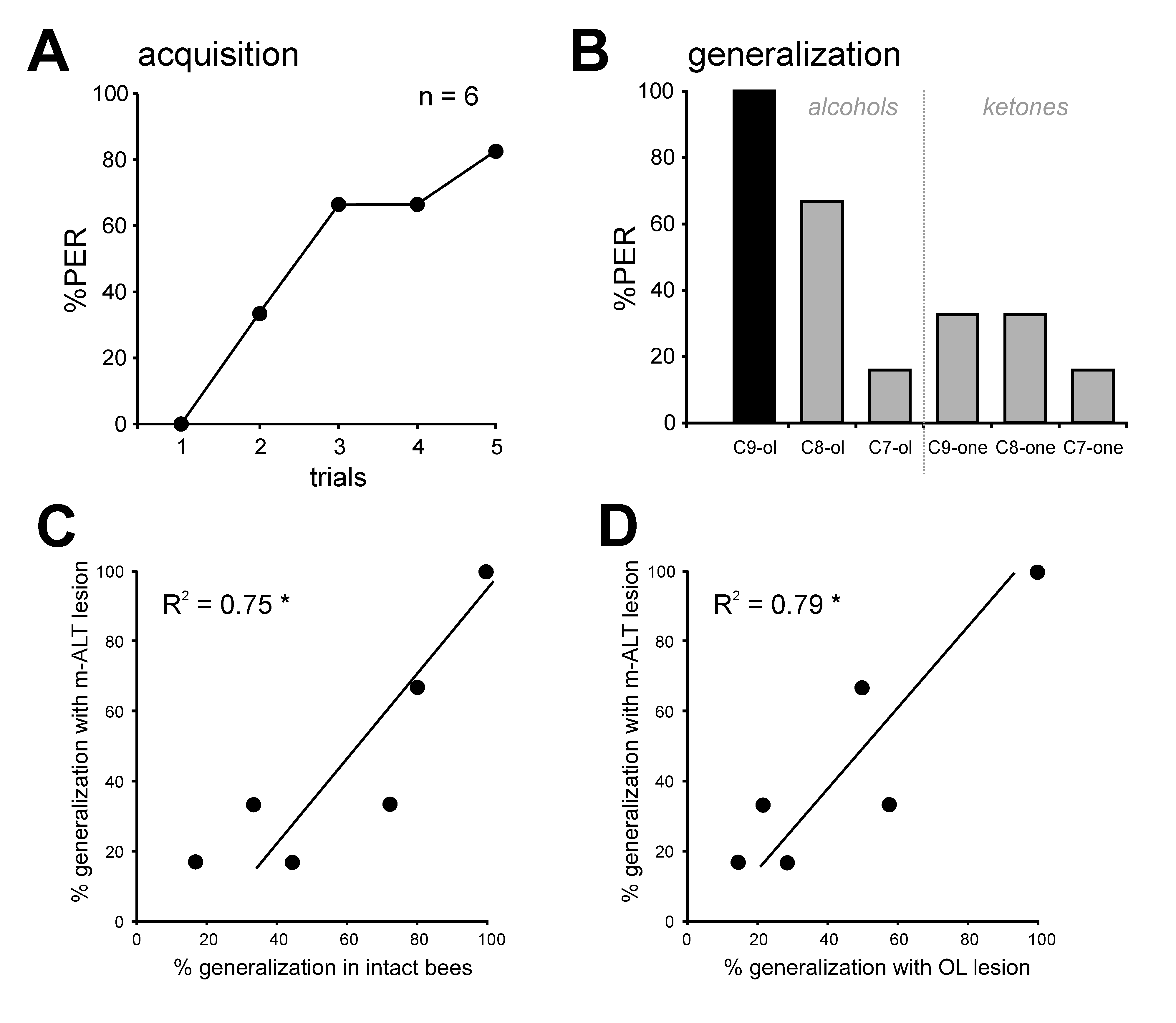

Supplement: Supplemental Figure 1 — PER conditioning and generalization performances in m-ALT lesioned bees, after selection of bees responding to the CS at the end of the generalization phase. (A) Acquisition performances (n = 6 bees). (B) Responses in the generalization tests. (C,D) Significant correlation between the responses of these m-ALT lesioned bees (n = 6) during generalization tests with those of intact bees (C, n = 36 bees, *p < 0.05, R2 = 0.75) and OL-lesioned bees (D, n = 14 bees, *p < 0.05, R2 = 0.79). [file Image1.TIF]
